# Socio-Demographic Inequalities in the Prevalence, Diagnosis and Management of Hypertension in India: Analysis of Nationally-Representative Survey Data

**DOI:** 10.1371/journal.pone.0086043

**Published:** 2014-01-23

**Authors:** Kath A. Moser, Sutapa Agrawal, George Davey Smith, Shah Ebrahim

**Affiliations:** 1 Faculty of Epidemiology and Population Health, London School of Hygiene and Tropical Medicine, London, United Kingdom; 2 South Asia Network for Chronic Disease, Public Health Foundation of India, New Delhi, India; 3 School of Social and Community Medicine, University of Bristol, Bristol, United Kingdom; University of Louisville, United States of America

## Abstract

**Background:**

Hypertension is a major contributing factor to the current epidemic of cardiovascular disease in India. Small studies suggest high, and increasing, prevalence especially in urban areas, with poor detection and management, but national data has been lacking. The aim of the current study was to use nationally-representative survey data to examine socio-demographic inequalities in the prevalence, diagnosis and management of hypertension in Indian adults.

**Methods:**

Using data on self-reported diagnosis and treatment, and blood pressure measurement, collected from 12,198 respondents aged 18+ in the 2007 WHO Study on Global Ageing and Adult Health in India, factors associated with prevalence, diagnosis and treatment of hypertension were investigated.

**Results:**

22% men and 26% women had hypertension; prevalence increased steeply with body mass index (<18.5 kg/m^2^: 18% men, 21% women; 25-29.9 kg/m^2^: 35% men, 35% women), was higher in the least poor vs. poorest (men: odds ratio (95%CI) 1.82 (1.20 to 2.76); women: 1.40 (1.08 to 1.81)), urban vs. rural men (1.64 (1.19 to 2.25)), and men recently vs. never using alcohol (1.96 (1.40 to 2.76)). Over half the hypertension in women, and 70% in men, was undetected with particularly poor detection rates in young urban men, and in poorer households. Two-thirds of men and women with detected hypertension were treated. Two-thirds of women treated had their hypertension controlled, irrespective of urban/rural setting or wealth. Adequate blood pressure control was sub-optimal in urban men.

**Conclusion:**

Hypertension is very common in India, even among underweight adults and those of lower socioeconomic position. Improved detection is needed to reduce the burden of disease attributable to hypertension. Levels of treatment and control are relatively good, particularly in women, although urban men require more careful attention.

## Introduction

Hypertension is a major contributing factor to the current epidemic of cardiovascular disease in India and many other low- and middle-income countries [1], [2]. The global burden of hypertension was estimated to be close to 1 billion adults in 2000, and predicted to increase to 1.56 billion by 2025 [3]. Worldwide, in excess of 7 million deaths annually may be attributable to hypertension [4] which is the third most important cause of the global burden of disease [5]. There is marked variation in levels of mean systolic blood pressure between countries, with highest levels evident in low- and middle-income countries, and a small decrease in mean systolic blood pressure globally since 1980, although the trends varied across regions [6]. Hypertension tends to be inversely related to socioeconomic position in high income countries [7] with the opposite often being the case in low- and middle-income countries [8]. Only in the later stages of the epidemiological transition does the burden of chronic disease including hypertension shift from higher to lower socioeconomic groups [2], [9]. A high and increasing prevalence of hypertension in both rural and urban areas of India has been reported in recent studies [10], [11] with higher prevalence in urban than rural areas and intermediate levels among migrants [12].

Detection and effective management of hypertension decreases the risk of stroke, myocardial infarction, chronic kidney disease and heart failure. Knowledge about the prevalence and social patterning of hypertension is essential for informing the public health effort to control hypertension in the community. In the US and other high-income countries in the 1970s what has become known as ‘the rule of halves’ found that only half of adults with hypertension were diagnosed, only half of those diagnosed were treated, and only half of those treated were well controlled [13], [14]. While detection and treatment has improved in many high-income countries over recent decades good control remains low [15], [16]. Recent small scale and local studies show that the rule of halves applies in India where detection, treatment and control of hypertension remain inadequate [11], [17]–[19]. However there is an urgent need for national data to confirm the situation. With the current epidemic in chronic disease, primarily affecting older people, the gap in the evidence base has become only too apparent. The nationally-representative surveys conducted in the WHO Study on Global Ageing and Adult Health (SAGE) programme (http://www.who.int/healthinfo/sage/cohorts/en/index2.html) are beginning to fill this gap by collecting detailed information on the health and well-being of adult populations and the ageing process [20], [21]. Using self-reports of hypertension diagnosis and treatment, in addition to blood pressure measurement, from the 2007 SAGE survey in India, we examined socio-demographic inequalities in the prevalence, diagnosis and management of hypertension in Indian adults and assessed ‘the rule of halves’ for detection, treatment and control.

## Methods

### Ethics statement

Ethical approval was not required for this analysis of anonymised secondary data. The 2007 SAGE Wave 1 survey of India received approval from the review board of the International Institute for Population Sciences in Mumbai, India. Respondents provided informed consent to participate in the survey. A standard consent form, approved by the World Health Organization ethics review committee, was read to the respondent in the respondent's language. If the respondent agreed to participate in the survey, and if s/he was literate, the form was provided to him/her to peruse and sign and was countersigned by the interviewer. If the respondent was illiterate and gave consent to participate, the interviewer confirmed this consent and signed on the form that the form had been read to the respondent, that s/he had understood the study and had agreed to participate. This procedure was approved by the review board of the International Institute for Population Sciences.

This analysis uses data collected in the 2007 SAGE Wave 1 survey of India (conducted by the International Institute for Population Sciences, Mumbai with the World Health Organization, Geneva). The SAGE survey took representative samples of six states in India (Assam, Karnataka, Maharashtra, Rajasthan, Uttar Pradesh and West Bengal) which can be modelled to a nationally representative sample. The survey consisted of a large sample of people aged 50 years and older and a smaller comparative sample aged 18–49 years, 12,198 respondents (4,717 men, 7,481 women) in total. The SAGE dataset is described in full elsewhere [20], [22] and the questionnaires can be found at http://www.who.int/healthinfo/sage/cohorts/en/index2.html (accessed 23 July 2013). SAGE Wave 1 data are available in the public domain at the same location.

The survey includes 3 types of information on hypertension.

1. Blood pressure (BP) measurement at physical examination, measured in the left wrist using a Boso Medistar Wrist Blood Pressure Monitor Model S (which avoids the need for different cuff sizes necessary with blood pressure measured in the upper arm). Validation studies of similar wrist blood pressure monitoring devices indicate they are capable of providing accurate measurements [23], [24] but that the position of the arm in relation to the heart is critical (http://www.bhsoc.org/bp-monitors/bp-monitors/). Respondents were asked to remain seated with legs uncrossed, positioning their arm level with their heart, taking 3 deep slow breaths before measurement started and then remaining relaxed and still while their BP was measured three times with at least one minute between each measurement [22].
2. Later in the interview respondents were asked: Have you ever been diagnosed with high BP (hypertension)?
3. Those answering ‘yes’ were asked: Have you been taking any medications or other treatment for it during a) the last 2 weeks? b) the last 12 months? (The questionnaire stated ‘Other treatment might include weight loss program or change in eating habits’).

We calculated mean systolic and diastolic BP using all three BP readings and where systolic > = 140 mmHg and/or diastolic > = 90 mmHg respondents were classified as having high BP at exam in accordance with guidelines [25]. We defined hypertension as high BP at exam and/or self-reported diagnosis; this included all those on treatment as only respondents reporting diagnosis were asked about treatment. Treatment was defined as being on medication or other treatment in the past 12 months. For respondents reporting diagnosis, or diagnosis and treatment, we examined whether their hypertension was controlled, defined as systolic BP <140 mmHg and diastolic <90 mmHg. The rule of halves for detection, treatment and control was assessed by splitting hypertension into the following proportions i) undiagnosed and uncontrolled (i.e. high BP at exam, but diagnosis not reported), ii) diagnosed but uncontrolled (i.e. high BP, diagnosis reported), and iii) controlled (i.e. diagnosis reported, BP not high). Both ii) and iii) were further sub-divided into those reporting being on treatment or not.

Socio-demographic and risk factors considered were respondents' area of residence (urban; rural); age; sex; religion (Hindu; Muslim; other religion); caste (scheduled tribe; scheduled caste; other backward caste; other); highest level of education completed (no formal education; less than primary school; primary school completed; secondary school completed; high school (or equivalent) completed; above high school); body mass index (BMI, <18.5, 18.5−, 23.0−, 25.0−, 30.0+ kg/m^2^ [26]) calculated from height and weight measurements where both values were non-missing; alcohol consumption (recent use (in last 30 days), used but not recently, never used); household wealth index (provided in dataset, derived using WHO standard approach to estimating permanent income from survey data on household ownership of durable goods, neighbourhood and dwelling characteristics, and access to water, sanitation, electricity etc [27]).

Survey response was high with 92% of the eligible persons contacted completing the interviews. Respondents with complete interviews and complete hypertension data were included in the analysis. Hypertension data was considered complete if the respondent had all three BP measurements and had answered yes or no when asked if ever diagnosed with high BP (hypertension). We examined whether respondents with complete hypertension data differed from those with incomplete hypertension data.

Outcome variables were mean systolic and diastolic BP, high BP at examination, self-reported diagnosis and treatment of hypertension, any indication of hypertension (that is, high BP at exam and/or self-reported diagnosis of hypertension), and control of hypertension. We explored the association between outcome variables and age, sex and other socio-demographic characteristics. Age-standardisation was conducted using the United Nations 2005 population of India (both sexes combined) as the standard [28]. Associations with socio-demographic characteristics were examined using linear regression for mean BP adjusted for age and socio-demographic variables, and logistic regression for the prevalence of hypertension adjusted for age. STATA statistical software version 10.0 was used. The analysis took account of the cluster sampling design. Supplied weighting factors were used throughout the analysis, including the regression analyses, to correct for the unequal probability of selection resulting from the sampling design. Detailed information on the survey weighting is available at http://apps.who.int/healthinfo/systems/surveydata/index.php/catalog/65#page=sampling&tab=study-desc. Numerators and denominators given in the text and tables refer to the unweighted sample.

## Results

Of the 12,198 survey respondents 12% (1,462) did not have complete interviews (494 partial interviews, 968 refusals, no contacts etc) and were excluded from further analysis. Of the remaining 10,736, a further 65 were excluded as they had incomplete hypertension data. Respondents with complete hypertension data were more likely to live in a rural area than the 65 with incomplete hypertension data (69.4% vs. 42.8%, p = 0.03). The mean age of those with complete hypertension data did not differ significantly from those with incomplete data, nor did the two groups differ in their composition by sex (p = 0.75), education level (p = 0.33) or household wealth (p = 0.74). In general, all further analyses were conducted on the 10,671 respondents (4,148 men, 6,523 women) with complete hypertension data. However as three further variables - household wealth index, caste, and BMI - contained missing data for 71, 63 and 137 respondents respectively, analyses including these variables were conducted on a slightly reduced (up to a maximum of 206) number of respondents. Study characteristics are shown in Table 1. The mean age was 41 years, with over two-thirds living in rural areas. One-third of either sex had a BMI below 18.5 kg/m^2^ while 24% of women and 21% of men had a BMI of 23 kg/m^2^ or above. Two-thirds of men and 99% of women reported never consuming alcohol.

**Table 1:** Characteristics of study population.

	MEN		WOMEN**		ALL	
	%	(n)	%	(n)	%	(n)
**total**	100.0	(4,148)	100.0	(6,523)	100.0	(10,671)
**age group**						
18–29	21.2	(257)	27.6	(1,270)	24.3	(1,527)
30–39	25.0	(345)	26.3	(1,248)	25.6	(1,593)
40–49	30.2	(394)	22.0	(951)	26.2	(1,345)
50–59	11.0	(1,342)	10.4	(1,481)	10.7	(2,823)
60–69	7.1	(1,098)	7.3	(1,010)	7.2	(2,108)
70+	5.6	(712)	6.5	(563)	6.0	(1,275)
mean age (95% CI)	42.0	(41.3, 42.8)	40.0	(39.5, 40.6)	41.1	(40.5, 41.6)
**place of residence**						
rural	70.0	(3,185)	68.8	(4,803)	69.4	(7,988)
urban	30.0	(963)	31.2	(1,720)	30.6	(2,683)
**highest education level**						
no education	21.1	(1,217)	49.6	(3,581)	35.0	(4,798)
<primary	9.4	(530)	7.5	(583)	8.5	(1,113)
primary	17.2	(730)	16.1	(904)	16.7	(1,634)
secondary	18.8	(667)	12.6	(681)	15.8	(1,348)
high school	20.3	(625)	10.0	(529)	15.3	(1,154)
>high school	13.4	(379)	4.2	(245)	8.9	(624)
**household wealth index** *						
Q1: poorest quintile	19.3	(693)	20.2	(1,167)	19.8	(1,860)
Q2	20.1	(801)	20.9	(1,227)	20.5	(2,028)
Q3	20.7	(780)	19.6	(1,249)	20.2	(2,029)
Q4	18.5	(896)	18.4	(1,357)	18.4	(2,253)
Q5: least poor quintile	21.4	(952)	20.9	(1,478)	21.2	(2,430)
**religion**						
hindu	83.7	(3,476)	85.1	(5,517)	84.4	(8,993)
muslim	12.7	(516)	11.8	(770)	12.3	(1,286)
other	3.6	(156)	3.1	(236)	3.4	(392)
**caste** *						
other	61.8	(2,486)	62.8	(3,764)	62.3	(6,250)
other backward caste	13.4	(632)	13.3	(1,133)	13.3	(1,765)
scheduled caste	18.9	(726)	17.6	(1,130)	18.3	(1,856)
scheduled tribe	5.9	(285)	6.4	(452)	6.1	(737)
**bmi** *						
<18.5 kg/m^2^	34.8	(1,441)	35.4	(2,211)	35.1	(3,652)
18.5–22.9	44.1	(1,816)	41.1	(2,585)	42.6	(4,401)
23–24.9	11.1	(417)	9.7	(614)	10.4	(1,031)
25–29.9	8.5	(369)	10.5	(770)	9.5	(1,139)
30+	1.6	(72)	3.3	(239)	2.4	(311)
**alcohol**						
never use	68.8	(2,923)	98.6	(6,371)	83.3	(9,294)
not recent use	15.1	(643)	0.6	(54)	8.0	(697)
recent use (in last 30 days)	16.2	(582)	0.9	(98)	8.7	(680)

percentages are weighted; n refers to number in unweighted sample.

* household wealth index missing for 71, caste missing for 63, BMI missing for 137 respondents.

** the sample includes a large number of women aged 18–49 years as part of a nested study.

In general, findings for mean diastolic BP mirrored those of systolic BP so only systolic BP data are shown in Table 2. Mean systolic BP increased with age for both sexes, with a steeper and smoother gradient in women than men. After full adjustment, mean systolic BP increased with BMI in both sexes, and recent users of alcohol had higher mean BP than never users. No wealth differentials were apparent in either sex. While no urban-rural differential was apparent in women, in men systolic BP was higher in urban than rural residents by 4.4 mmHg which attenuated after full adjustment to 3.2 mmHg.

**Table 2:** Mean systolic blood pressure (mm Hg) by sociodemographic characteristics.

	MEN				WOMEN			
	unadjusted		full adj* (excl caste)		unadjusted		full adj* (excl caste)	
	mean	(95% CI)	diff from	(95% CI)	mean	(95% CI)	diff from	(95% CI)
	systolic BP		baseline		systolic BP		baseline	
**age group**								
18–29	114.8	(113.4, 116.2)	ref		110.7	(109.4, 112.0)	ref	
30–39	117.3	(115.6, 119.0)	1.0	(−1.0, 3.1)	113.7	(112.6, 114.9)	1.3	(−0.5, 3.1)
40–49	117.1	(114.9, 119.3)	1.6	(−0.8, 4.0)	118.0	(116.6, 119.5)	**5.2**	**(3.1, 7.3)**
50–59	120.4	(119.1, 121.8)	**5.1**	**(3.3, 6.9)**	123.0	(121.4, 124.6)	**10.1**	**(7.8, 12.3)**
60–69	122.2	(120.3, 124.1)	**7.1**	**(4.6, 9.6)**	127.3	(125.7, 129.0)	**15.2**	**(12.8, 17.5)**
70+	125.4	(122.7, 128.0)	**11.0**	**(8.1, 13.9)**	129.2	(126.8, 131.5)	**16.8**	**(14.1, 19.6)**
age effect per 10 years	1.84	(1.39, 2.30)			3.80	(3.45, 4.15)		
**place of residence**								
rural	116.5	(115.6, 117.5)	ref		116.7	(115.9, 117.4)	ref	
urban	120.9	(118.8, 123.0)	**3.2**	**(0.9, 5.5)**	117.0	(115.3, 118.8)	−0.1	(−1.8, 1.7)
age effect per 10 years: rural	1.72	(1.20, 2.23)			3.65	(3.28, 4.01)		
age effect per 10 years: urban	2.25	n/a**			4.16	n/a**		
**highest education level**								
no education	118.0	(116.2, 119.9)	ref		118.6	(117.6, 119.6)	ref	
<primary	118.5	(116.0, 121.0)	1.0	(−1.9, 3.9)	117.0	(115.1, 118.9)	−0.4	(−2.4, 1.6)
primary	118.4	(116.0, 120.7)	−0.2	(−3.0, 2.6)	117.2	(115.1, 119.2)	1.0	(−0.9, 3.0)
secondary	115.9	(114.0, 117.8)	−1.9	(−4.4, 0.6)	114.4	(112.8, 116.0)	0.6	(−1.3, 2.4)
high school	117.1	(115.0, 119.3)	−2.1	(−4.8, 0.7)	112.3	(110.2, 114.5)	−2.0	(−4.5, 0.6)
>high school	120.2	(117.6, 122.7)	−0.2	(−3.2, 2.7)	110.6	(107.7, 113.6)	−**4.2**	**(**−**7.4,** −**1.1)**
**household wealth index**								
Q1: poorest quintile	115.8	(113.4, 118.1)	ref		117.1	(115.5, 118.7)	ref	
Q2	116.7	(114.7, 118.7)	0.1	(−2.6, 2.8)	117.4	(115.6, 119.2)	−0.6	(−2.5, 1.4)
Q3	117.6	(115.7, 119.6)	0.3	(−2.6, 3.3)	115.7	(114.3, 117.1)	−**2.6**	**(**−**4.7,** −**0.5)**
Q4	117.8	(115.7, 119.9)	−0.9	(−4.0, 2.2)	116.3	(114.9, 117.6)	−1.9	(−3.9, 0.0)
Q5: least poor quintile	121.0	(119.3, 122.8)	1.0	(−2.2, 4.1)	117.3	(116.0, 118.6)	−1.7	(−4.0, 0.6)
**religion**								
hindu	118.2	(117.2, 119.2)	ref		116.5	(115.8, 117.3)	ref	
muslim	116.1	(113.6, 118.7)	−1.5	(−4.0, 1.0)	117.2	(114.9, 119.5)	0.4	(−1.9, 2.7)
other	115.6	(110.1, 121.1)	−2.1	(−7.3, 3.2)	122.2	(118.6, 125.9)	**5.2**	**(1.4, 9.1)**
**caste**								
other	118.1	(116.8, 119.3)			116.7	(115.8, 117.7)		
other backward caste	119.7	(117.3, 122.1)			116.5	(115.2, 117.8)		
scheduled caste	115.5	(113.4, 117.6)			116.2	(114.7, 117.6)		
scheduled tribe	119.1	(116.0, 122.2)			118.7	(116.1, 121.2)		
**bmi**								
<18.5 kg/m^2^	112.7	(111.4, 113.9)	ref		113.9	(112.7, 115.0)	ref	
18.5–22.9	118.4	(117.2, 119.6)	**5.8**	**(3.9, 7.7)**	116.7	(115.6, 117.7)	**3.6**	**(2.4, 4.7)**
23–24.9	123.7	(120.8, 126.5)	**11.5**	**(8.7, 14.4)**	119.3	(117.0, 121.6)	**6.4**	**(4.0, 8.9)**
25–29.9	126.9	(123.1, 130.7)	**14.2**	**(10.0, 18.4)**	122.7	(120.7, 124.8)	**9.0**	**(6.6, 11.4)**
30+	122.4	(116.0, 128.8)	**10.4**	**(3.6, 17.1)**	123.9	(120.5, 127.3)	**10.0**	**(6.3, 13.7)**
**alcohol use**								
never use	117.1	(116.1, 118.1)	ref		116.7	(115.9, 117.4)	ref	
not recent use	118.6	(116.4, 120.9)	1.2	(−1.0, 3.5)	123.0	(116.8, 129.3)	4.4	(−1.4, 10.3)
recent use (in last 30 days)	120.3	(117.7, 122.9)	**4.1**	**(1.6, 6.7)**	123.8	(118.4, 129.2)	**6.4**	**(1.2, 11.7)**

* adjusted for all variables in the table, with the exception of caste.

** standard errors not available because one stratum contained only a single sampling unit.

The age-standardised prevalence of hypertension was 23% in men and 26% in women increasing, in both sexes, with age from 13% under age 30 to over 40% at ages 70 and over (Table 3). In men, hypertension was more prevalent in urban than rural areas (age-standardised prevalence, 28% vs. 21%) with the age-adjusted odds of men in urban areas having hypertension 1.70 (95%CI 1.21 to 2.38) times that of men in rural areas. In both sexes the odds of having hypertension was higher in the least poor as compared with the poorest wealth quintile, and increased steeply with BMI. Although hypertension was more prevalent in overweight and less poor groups, even groups with very low BMI and groups in the poorest wealth category had appreciable levels of hypertension (BMI<18.5: 18% men, 21% women; poorest: 20% men, 24% women). In men, the odds of having hypertension was high in recent (2.01 (95%CI 1.40 to 2.89)) and past (1.58 (95%CI 1.10 to 2.25)) users of alcohol compared with never users.

**Table 3:** Prevalence of hypertension* by sociodemographic characteristics.

	MEN				WOMEN			
	no. with	crude (age-standardised)	odds ratios		no. with	crude (age-standardised)	odds ratios	
	hypertension	prevalence %	age adjusted	(95% CI)	hypertension	prevalence %	age adjusted	(95% CI)
**total**	1,390	25.2 (22.9)			2,030	27.1 (25.7)		
**age group**								
18–29	38	12.3			161	13.0		
30–39	82	24.6			268	22.0		
40–49	101	27.4			314	34.1		
50–59	472	32.0			571	39.5		
60–69	392	31.5			440	43.5		
70+	305	42.5			276	45.2		
**place of residence**								
rural	992	22.5 (21.2)	1.00		1,407	26.1 (25.0)	1.00	
urban	398	31.4 (27.9)	**1.70**	**(1.21, 2.38)**	623	29.2 (27.2)	1.16	(0.93, 1.44)
**highest education level**								
no education	383	24.0 (23.0)	1.00		1,188	30.1 (26.1)	1.00	
<primary	178	25.3 (23.4)	1.07	(0.66, 1.72)	184	25.2 (24.2)	0.94	(0.70, 1.26)
primary	232	28.6 (28.8)	1.48	(0.98, 2.24)	267	27.5 (27.7)	1.19	(0.92, 1.55)
secondary	223	21.2 (20.2)	1.03	(0.67, 1.59)	188	21.0 (28.3)	1.10	(0.78, 1.56)
high school	220	23.5 (22.2)	1.25	(0.82, 1.92)	146	22.1 (28.9)	1.23	(0.86, 1.77)
>high school	154	30.6 (31.2)	**1.96**	**(1.21, 3.16)**	57	22.8 (26.2)	1.19	(0.75, 1.90)
**household wealth index**								
Q1: poorest quintile	188	21.4 (19.5)	1.00		311	24.2 (24.4)	1.00	
Q2	237	21.3 (22.5)	1.01	(0.65, 1.59)	339	25.5 (23.8)	1.07	(0.76, 1.49)
Q3	250	25.7 (21.3)	1.36	(0.88, 2.11)	378	25.9 (25.3)	1.13	(0.86, 1.47)
Q4	335	26.0 (22.6)	1.26	(0.81, 1.96)	442	28.7 (27.2)	1.30	(0.97, 1.73)
Q5: least poor quintile	373	31.4 (30.6)	**1.95**	**(1.27, 3.00)**	543	30.9 (28.4)	**1.40**	**(1.07, 1.84)**
**religion**								
hindu	1,182	25.8 (23.3)	1.00		1,662	26.3 (24.9)	1.00	
muslim	147	21.5 (19.6)	0.79	(0.49, 1.28)	278	30.2 (29.5)	1.28	(0.95, 1.72)
other	61	24.0 (24.0)	0.93	(0.48, 1.82)	90	36.1 (31.6)	**1.62**	**(1.02, 2.56)**
**caste**								
other	795	25.1 (22.6)	1.00		1,210	28.2 (26.7)	1.00	
other backward caste	266	29.0 (25.5)	1.24	(0.85, 1.80)	376	25.1 (23.8)	0.87	(0.71, 1.06)
scheduled caste	213	20.6 (19.8)	0.81	(0.55, 1.19)	303	24.6 (23.6)	0.86	(0.67, 1.10)
scheduled tribe	108	32.5 (29.8)	1.52	(0.90, 2.57)	130	26.3 (25.3)	0.95	(0.66, 1.38)
**bmi**								
<18.5 kg/m^2^	370	18.8 (18.1)	1.00		527	21.6 (20.6)	1.00	
18.5−	595	23.3 (20.9)	1.39	(1.00, 1.95)	771	25.4 (24.9)	**1.28**	**(1.05, 1.56)**
23.0−	186	33.8 (32.2)	**2.42**	**(1.51, 3.86)**	223	34.1 (31.5)	**1.86**	**(1.36, 2.54)**
25.0−	185	46.6 (36.7)	**3.78**	**(2.35, 6.09)**	356	38.7 (34.9)	**2.03**	**(1.55, 2.65)**
30.0+	39	37.1 (26.1)	**2.88**	**(1.35, 6.15)**	120	48.7 (40.0)	**3.17**	**(1.98, 5.07)**
**alcohol use**								
never use	915	21.3 (19.7)	1.00		1,968	26.9 (25.7)	1.00	
not recent use	234	31.1 (26.5)	**1.58**	**(1.10, 2.25)**	20	37.7 (25.0)	1.16	(0.55, 2.46)
recent use (in last 30 days)	241	36.2 (32.3)	**2.01**	**(1.40, 2.89)**	42	35.5 (24.9)	1.26	(0.66, 2.39)

* defined as > = 140/90 mmHg and/or diagnosis.

**Figure 1:**
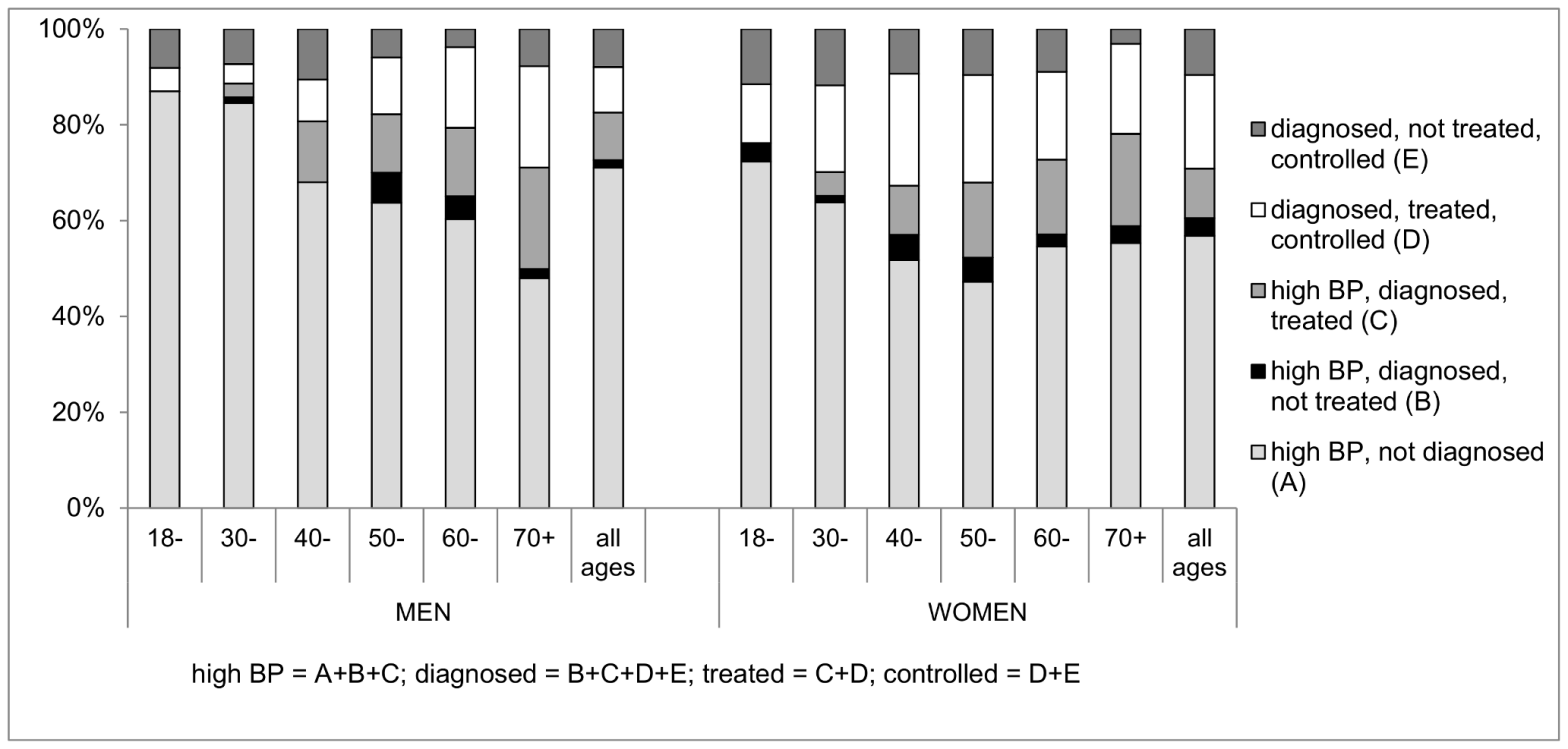
Percentage of hypertension that is diagnosed, treated, controlled, by age and sex.

Table 4 shows the prevalence, diagnosis, treatment and control of hypertension by age and sex, with the rule of halves findings summarised in Figure 1. In both sexes, the age-standardised prevalence of high BP at examination was around 19%, with the rate increasing from 10% under age 30 to around one-third at ages 70 and over. The proportion of men with high BP at exam doubled between the 18–29 and 30–39 age groups, then remained at 20–25% through the 30 s to 60 s. Only a small proportion of those with high BP at exam reported being diagnosed with hypertension, with proportions particularly low at younger ages; none of the men under 30 with high BP, and only 4% of those in their 30 s, reported being diagnosed. The situation was slightly better among women, but in no age group in either sex did the proportion of those with high BP reporting diagnosis exceed one-third; this indicates detection rates far worse than the 50% suggested by the rule of halves. Among both men and women reporting diagnosis with hypertension, two-thirds reported treatment in the past 12 months; half the men and two-thirds of the women treated had their BP controlled.

**Table 4:** Prevalence, diagnosis, treatment and control of hypertension by age and sex.

	MEN								WOMEN							
							**crude (age-stan-**								**crude (age-stan-**	
age group	18–29	30–39	40–49	50–59	60–69	70+	**dardised) rate**	*n*	18–29	30–39	40–49	50–59	60–69	70+	**dardised) rate**	*n*
*n*	*257*	*345*	*394*	*1,342*	*1,098*	*712*		***4,148***	*1,270*	*1,248*	*951*	*1,481*	*1,010*	*563*		***6,523***
**Reporting diagnosis of hypertension (%)**	1.6	3.8	8.8	11.7	12.4	22.1	**7.3 (6.2)**	*510*	3.6	8.0	16.5	20.8	19.7	20.2	**11.6 (10.9)**	*894*
*of those reporting diagnosis, % reporting treatment in past 12 months*	38	46	67	66	79	81	**67**	*388*	48	63	70	72	75	85	**70**	*675*
*of those reporting diagnosis & treatment, % controlled at exam (<140/<90)*	100	59	41	49	54	50	**49**	*197*	95	79	69	59	54	49	**66**	*402*
**High BP at examination +140/+90 (%)**	10.7	21.8	22.2	26.3	25.0	30.2	**20.8 (19.0)**	*1,114*	9.9	15.5	23.0	26.9	31.7	35.3	**19.2 (18.3)**	*1,472*
*of those with high BP at exam, % reporting diagnosis*	0	4	16	23	24	32	**14**	*234*	5	9	23	30	25	29	**20**	*336*
*of those with high BP at exam, % reporting diagnosis & treatment*	0	3	16	15	18	30	**12**	*191*	1	7	15	23	22	25	**14**	*273*
**Any indication of hypertension (%)**	12.3	24.6	27.4	32.0	31.5	42.5	**25.2 (22.9)**	*1,390*	13.0	22.0	34.1	39.5	43.5	45.2	**27.1 (25.7)**	*2,030*
*of those with any indication of hyp, % reporting diagnsosis*	13	15	32	36	40	52	**29**	*510*	28	36	48	53	45	45	**43**	*894*

percentages are weighted; n refers to number in unweighted sample.

subgroups are not mutually exclusive.

### Urban-rural differences

**Figure 2:**
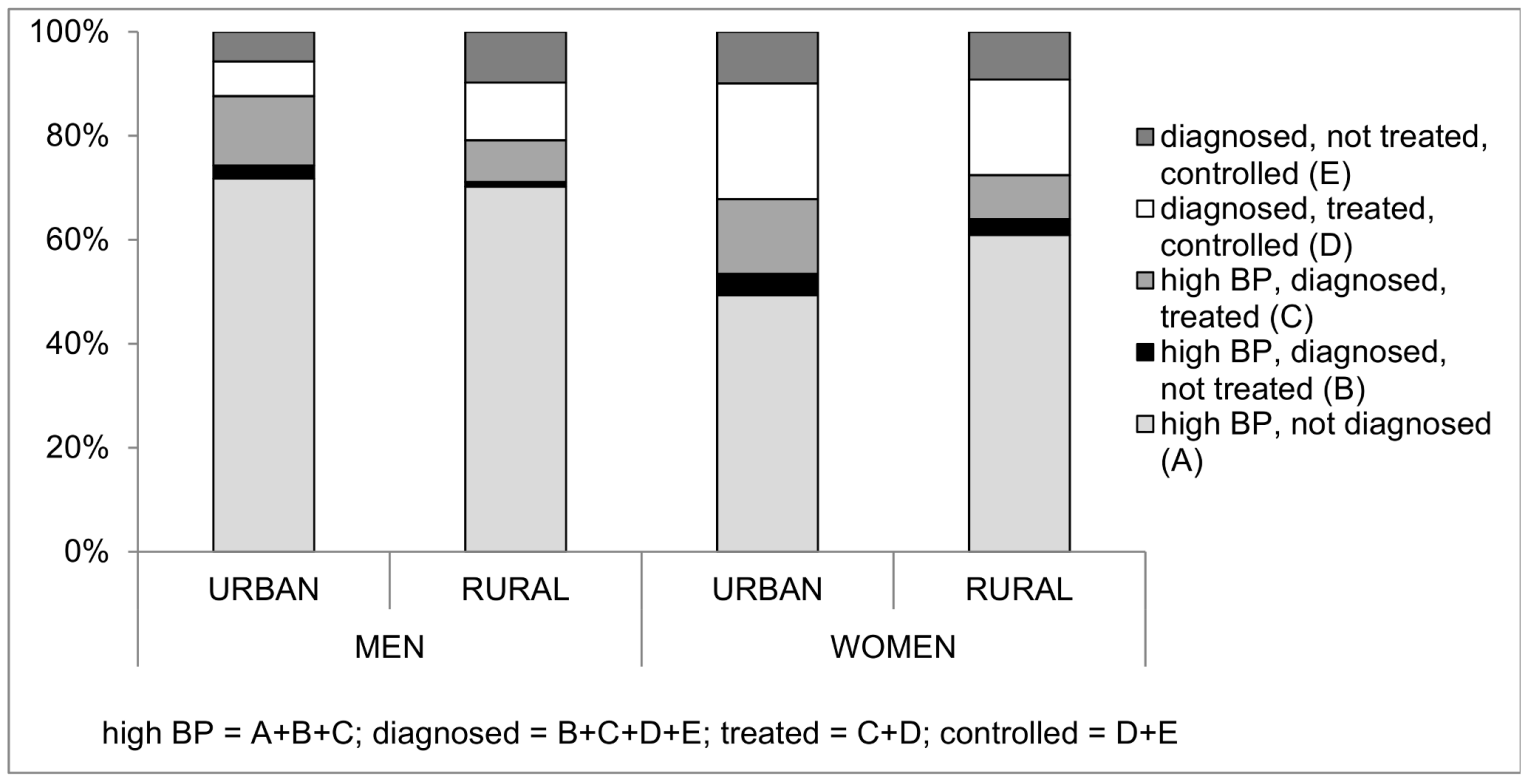
Percentage of hypertension that is diagnosed, treated, controlled, by urban-rural residence & sex.

One-quarter of urban men and 17% of rural men had high BP at examination, with no urban-rural differential observed in women (18%) (Table 5). The expected increase in prevalence of high BP with age was not apparent for urban men; a very high proportion, 30–40%, of those in their 30 s and 40 s had high BP, although these figures were based on relatively small numbers. A greater proportion of those with high BP reported diagnosis in urban than rural areas (men: 18% urban, 11% rural; women: 27%, 16%). Among men there was no urban-rural differential in the proportion of those with any indication of hypertension who were diagnosed, although in both settings, at around 30%, detection rates were low (Figure 2). The situation was better for women. In either sex two-thirds of those diagnosed were treated, with little evidence of an urban-rural differential. Control among those treated was sub-optimal in urban men, only one-third of whom had their BP controlled.

**Table 5:** Prevalence, diagnosis, treatment and control of hypertension by urban-rural residence, age and sex.

	URBAN								RURAL							
MEN							**crude (age-stan-**								**crude (age-stan-**	
age group	18–29	30–39	40–49	50–59	60–69	70+	**dardised) rate**	*n*	18–29	30–39	40–49	50–59	60–69	70+	**dardised) rate**	*n*
*n*	*72*	*76*	*75*	*330*	*242*	*168*		***963***	*185*	*269*	*319*	*1,012*	*856*	*544*		***3,185***
**Reporting diagnosis of hypertension (%)**	0.0	2.8	12.8	14.3	14.8	36.4	**8.8 (7.4)**	*173*	2.6	4.2	7.2	10.5	11.4	16.2	**6.7 (5.8)**	*337*
*of those reporting diagnosis, % reporting treatment in past 12 months*	-	0	70	64	84	94	**71**	*146*	38	58	65	67	76	70	**64**	*242*
*of those reporting diagnosis & treatment, % controlled at exam (<140/<90)*	-	0	13	44	62	43	**34**	*77*	100	59	62	52	49	59	**59**	*120*
**High BP at examination +140/+90 (%)**	8.7	31.8	40.4	30.1	20.6	37.2	**27.6 (24.5)**	*305*	11.9	18.0	15.2	24.7	26.9	27.3	**17.8 (17.1)**	*809*
*of those with high BP at exam, % reporting diagnosis*	0	3	19	34	27	53	**18**	*80*	0	5	12	17	23	21	**11**	*154*
*of those with high BP at exam, % reporting diagnosis & treatment*	0	0	19	17	23	52	**15**	*69*	0	5	12	14	17	17	**10**	*122*
**Any indication of hypertension (%)**	8.7	33.7	45.4	34.3	29.7	54.0	**31.4 (27.9)**	*398*	14.4	21.2	20.6	31.1	32.2	37.7	**22.5 (21.2)**	*992*
*of those with any indication of hyp, % reporting diagnsosis*	0	8	28	42	50	67	**28**	*173*	18	20	35	34	36	43	**30**	*337*
																
WOMEN							**crude (age-stan-**								**crude (age-stan-**	
age group	18–29	30–39	40–49	50–59	60–69	70+	**dardised) rate**	*n*	18–29	30–39	40–49	50–59	60–69	70+	**dardised) rate**	*n*
*n*	*314*	*316*	*259*	*397*	*268*	*166*		***1,720***	*956*	*932*	*692*	*1,084*	*742*	*397*		***4,803***
**Reporting diagnosis of hypertension (%)**	2.9	8.7	18.5	32.6	25.0	34.3	**14.7 (13.6)**	*349*	3.9	7.7	15.5	15.7	17.4	13.7	**10.2 (9.7)**	*545*
*of those reporting diagnosis, % reporting treatment in past 12 months*	66	71	71	73	64	85	**73**	*278*	43	59	69	71	83	86	**68**	*397*
*of those reporting diagnosis & treatment, % controlled at exam (<140/<90)*	100	70	55	64	74	42	**61**	*170*	92	85	78	55	44	58	**69**	*232*
**High BP at examination +140/+90 (%)**	6.5	19.4	24.7	29.7	25.7	36.2	**19.8 (18.3)**	*405*	11.3	13.7	22.1	25.6	34.3	34.9	**18.9 (18.2)**	*1,067*
*of those with high BP at exam, % reporting diagnosis*	0	13	30	40	22	57	**27**	*131*	6	7	19	26	26	16	**16**	*205*
*of those with high BP at exam, % reporting diagnosis & treatment*	0	10	24	29	16	47	**21**	*108*	1	5	11	20	23	14	**11**	*165*
**Any indication of hypertension (%)**	9.3	25.6	35.8	50.5	45.1	49.8	**29.2 (27.2)**	*623*	14.6	20.4	33.2	34.7	42.8	43.1	**26.1 (25.0)**	*1,407*
*of those with any indication of hyp, % reporting diagnsosis*	31	34	52	64	55	69	**50**	*349*	27	38	47	45	41	32	**39**	*545*

percentages are weighted; n refers to number in unweighted sample.

subgroups are not mutually exclusive.

### Wealth differences

**Figure 3:**
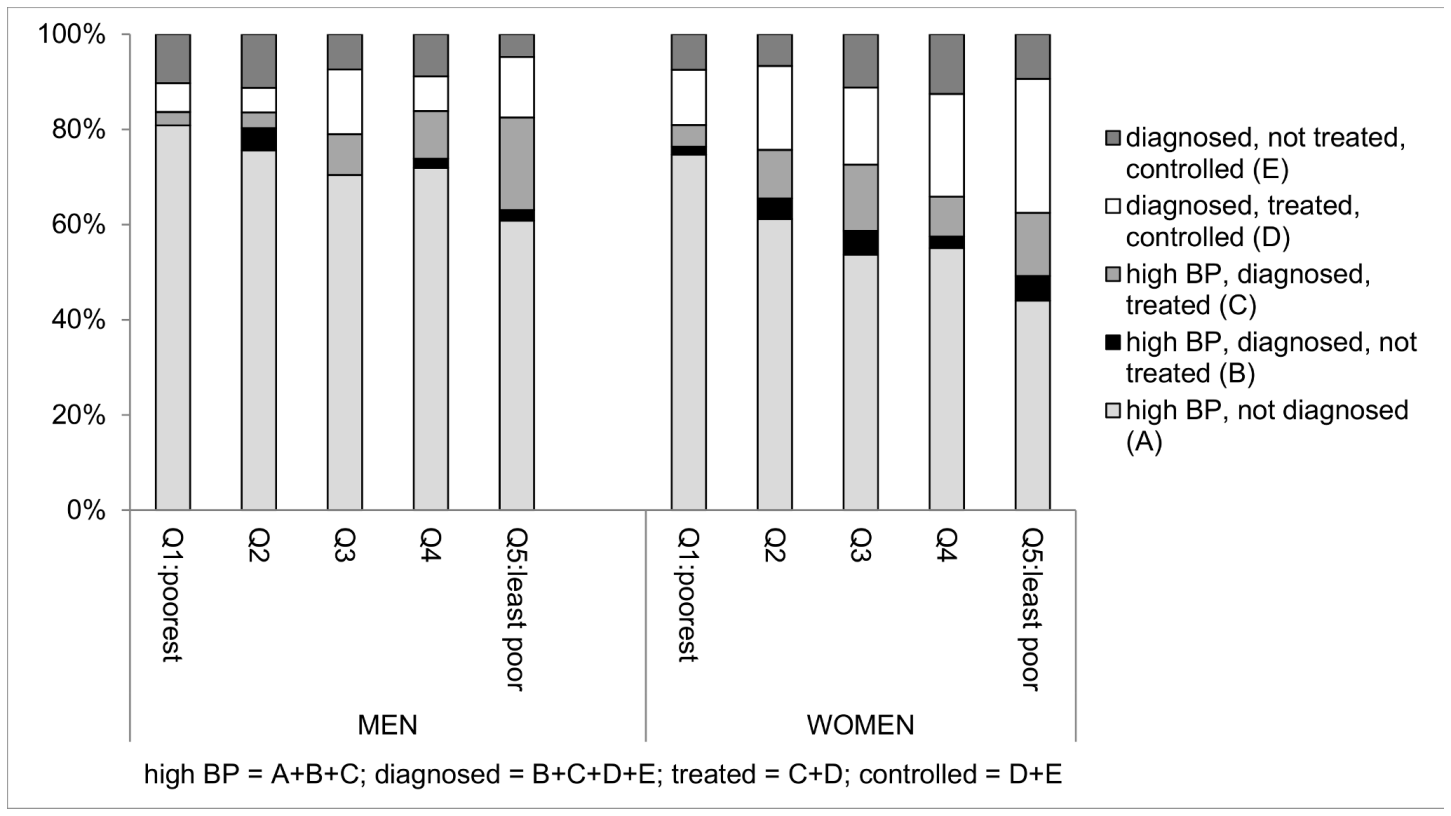
Percentage of hypertension that is diagnosed, treated, controlled, by household wealth & sex.

In both sexes, the proportion reporting diagnosis of hypertension showed marked positive gradients from poorest to least poor quintiles of household wealth (Table 6). The prevalence of high BP at examination in men showed a less pronounced gradient and among women there was no gradient with household wealth. However, of those with high BP at exam the proportion reporting diagnosis increased steeply with wealth (poorest: 3% men, 7% women; least poor: 26%, 29%). When considering any indication of hypertension, the proportion reporting diagnosis was twice as high in the least poor as the poorest quintile (39% vs. 19% men; 56% vs. 25% women) (Table 6, Figure 3), but, excepting women in less poor households, detection rates were low. Two-thirds of women reporting diagnosis were treated, and around two-thirds of those treated had adequate BP control, regardless of household wealth. Treatment rates in men increased with household wealth, although control among those treated appeared better in poorer quintiles.

**Table 6:** Prevalence, diagnosis, treatment and control of hypertension by wealth quintile and sex.

	Q1: POOREST		Q2		Q3		Q4		Q5: LEAST POOR	
MEN	**crude (age-stan-**		**crude (age-stan-**		**crude (age-stan-**		**crude (age-stan-**		**crude (age-stan-**	
	**dardised) rate**	*n*	**dardised) rate**	*n*	**dardised) rate**	*n*	**dardised) rate**	*n*	**dardised) rate**	*n*
		***693***		***801***		***780***		***896***		***952***
**Reporting diagnosis of hypertension (%)**	**4.1 (3.8)**	*41*	**5.1 (5.3)**	*61*	**7.7 (6.8)**	*77*	**7.3 (5.5)**	*140*	**12.2 (10.8)**	*190*
*of those reporting diagnosis, % reporting treatment in past 12 months*	**46**	*30*	**36**	*34*	**75**	*63*	**62**	*101*	**83**	*159*
*of those reporting diagnosis & treatment, % controlled at exam (<140/<90)*	**70**	*14*	**61**	*20*	**62**	*31*	**41**	*52*	**40**	*79*
**High BP at examination +140/+90 (%)**	**17.9 (16.3)**	*164*	**17.8 (19.2)**	*200*	**20.3 (16.3)**	*208*	**21.8 (19.3)**	*262*	**25.9 (25.5)**	*274*
*of those with high BP at exam, % reporting diagnosis*	**3**	*17*	**9**	*24*	**11**	*35*	**14**	*67*	**26**	*91*
*of those with high BP at exam, % reporting diagnosis & treatment*	**3**	*16*	**4**	*14*	**11**	*32*	**12**	*49*	**24**	*80*
**Any indication of hypertension (%)**	**21.4 (19.5)**	*188*	**21.3 (22.5)**	*237*	**25.7 (21.3)**	*250*	**26.0 (22.6)**	*335*	**31.4 (30.6)**	*373*
*of those with any indication of hyp, % reporting diagnsosis*	**19**	*41*	**24**	*61*	**30**	*77*	**28**	*140*	**39**	*190*
										
WOMEN	**crude (age-stan-**		**crude (age-stan-**		**crude (age-stan-**		**crude (age-stan-**		**crude (age-stan-**	
	**dardised) rate**	*n*	**dardised) rate**	*n*	**dardised) rate**	*n*	**dardised) rate**	*n*	**dardised) rate**	*n*
		***1,167***		***1,227***		***1,249***		***1,357***		***1,478***
**Reporting diagnosis of hypertension (%)**	**6.1 (5.9)**	*77*	**9.8 (8.4)**	*117*	**12.1 (11.7)**	*168*	**12.8 (12.3)**	*225*	**17.3 (15.9)**	*304*
*of those reporting diagnosis, % reporting treatment in past 12 months*	**64**	*54*	**72**	*88*	**65**	*118*	**67**	*166*	**74**	*246*
*of those reporting diagnosis & treatment, % controlled at exam (<140/<90)*	**72**	*35*	**63**	*46*	**54**	*61*	**72**	*108*	**68**	*150*
**High BP at examination +140/+90 (%)**	**19.5 (19.9)**	*257*	**19.3 (18.6)**	*273*	**18.8 (18.4)**	*285*	**18.9 (17.9)**	*289*	**19.3 (17.6)**	*353*
*of those with high BP at exam, % reporting diagnosis*	**7**	*23*	**19**	*51*	**26**	*75*	**16**	*72*	**29**	*114*
*of those with high BP at exam, % reporting diagnosis & treatment*	**6**	*19*	**13**	*42*	**19**	*57*	**13**	*58*	**21**	*96*
**Any indication of hypertension (%)**	**24.2 (24.4)**	*311*	**25.5 (23.8)**	*339*	**25.9 (25.3)**	*378*	**28.7 (27.2)**	*442*	**30.9 (28.4)**	*543*
*of those with any indication of hyp, % reporting diagnsosis*	**25**	*77*	**39**	*117*	**47**	*168*	**45**	*225*	**56**	*304*

percentages are weighted; n refers to number in unweighted sample.

subgroups are not mutually exclusive.

## Discussion

These analyses indicate that over one-fifth of Indian adults had hypertension, most of which - half in women, 70% in men - went undetected and therefore remained untreated and uncontrolled. Less than one-quarter of adults with measured high BP were aware of their condition, with diagnosis rates at younger ages particularly bad. On the other hand the data suggest that of the hypertension that was detected, treatment rates, and control among those treated, generally exceeded 50% which, although sub-optimal, is comparable with the situation in more affluent countries [15], [16].

There were differentials across population groups. Although hypertension was particularly prevalent in the least poor and the overweight, prevalence in the poor and the underweight was also high. These findings do not support the idea that hypertension is a condition only of affluence [29] and therefore of low priority for health and development programmes in India and other low- and middle-income countries. The lack of the usual age gradient in the prevalence of measured high BP in men is surprising. In our study recent alcohol use was much higher in underweight than overweight men, in rural than urban areas, and highest among men in their 30 s and 40 s. This, and other recent exposures such as increased dietary salt intake and low levels of physical activity in younger people, may be contributing to our findings [30], [31]. There were important differences between the sexes. The higher overall rates of diagnosis seen in women, especially those in urban locations and in better off households, probably result from contact with health services around childbearing. National Family Health Survey data for 2005–06 indicate that 77% of women attended an antenatal clinic for their most recent birth, and 64% of attendees had their BP measured [32]; attendance was much less common in rural than urban areas, and in poorer households, as was the proportion of attendees who had their BP measured. Our data show for women, but not men, a lack of patterning of treatment and control of hypertension across urban-rural or household wealth categories suggesting that the quality of care is more of an issue for men than women. The failure to detect hypertension in younger men, and the poor control in urban men, is of particular importance, suggesting a need for more proactive surveillance.

These analyses, based on recent nationally-representative survey data, provide up to date national estimates of the prevalence, diagnosis, treatment and control of hypertension amongst adults in India. This is important new information as, to date, most evidence on hypertension, and chronic disease more generally, in India has come from small scale and/or local studies.

The SAGE survey has the strength of containing blood pressure measurements and self-reported information on diagnosis and treatment of hypertension thus providing the opportunity to compare and contrast clinical measurements with self-reported data. The accuracy and reliability of Boso BP instruments, widely used in Germany, has been recognised by the German consumer safety organisation with the Medistar S coming top in the wrist category and the German Hypertension Society also confirms their high accuracy [33]. Self-reported information may be affected by recall biases and reporting errors which may vary by socio-demographic characteristics. As the survey question on treatment did not differentiate taking medication, weight loss program or change in eating habits, it was not possible to identify the subgroup taking hypertension medication, as recommended by the JNC guidelines as a diagnostic criteria for hypertension [25]. The adequacy of assessing prevalence of high BP using measurements made at a single clinic visit has been questioned [34] as it does not mirror clinical guidance of making repeated measurements over several weeks in order to avoid regression to the mean effects. These effects lead to over-estimation of the prevalence of high BP as the within-person fluctuations in blood pressure will tend to underestimate the “usual” level of blood pressure [35]. However, measurements made on a single occasion do have strong predictive power for cardiovascular disease in epidemiological studies [36], [37].

This nationally-representative study shows high rates of hypertension in adults in India affecting both the poor and the better off and with sub-optimal detection particularly among young urban men and poorer households. Tackling low detection rates is the priority as, once detected, the results indicate that rates of treatment and control of hypertension are relatively good. Substantial effort – both in improving clinical practice and in preventing high BP in the first place - is needed to reduce the high rates of hypertension, and the resulting large burden of cardiovascular disease, in India.
